# Life history of a new Paraceratheriid from the Early Oligocene of Northwest China

**DOI:** 10.1038/s41598-025-13365-w

**Published:** 2025-08-06

**Authors:** Xiaokang Lu, Tao Deng

**Affiliations:** 1 Department of Human Anatomy, Henan University of Chinese Medicine, Zhengzhou, 450046 Henan China; 2 Institute of Vertebrate Paleontology and Paleoanthropology, Chinese Academy of Sciences, Beijing, 100044 China; 3 University of Chinese Academy of Sciences, Beijing, 100049 China

**Keywords:** Evolution, Zoology, Biogeochemistry

## Abstract

Understanding life history is crucial for elucidating mammalian evolution. Body size and tooth development have long been fundamental parameters in reconstructing the life histories of both fossil and extant species. The superfamily Rhinocerotoidea exhibits a protracted evolutionary history from the Eocene to the present, with its large body sizes and high-crowned teeth providing compelling evidence for such studies. In this research, we describe *Turpanotherium qiui* sp. nov., a new species within the family Paraceratheriidae, identified from a mandible recovered from the Early Oligocene of Northwest China. Analysis of elemental concentrations revealed insights into both longitudinal life history patterns and discrete developmental events. Significant variations in barium and strontium concentrations in the enamel were associated with neonatal development and breastfeeding phases, whereas zinc, strontium, and barium concentrations in the dentine displayed marked seasonal variability. Analysis of Retzius lines in enamel and annual lines in cementum from tooth histology reveals that this Paleogene species exhibited a life history pace comparable to its Quaternary relatives. The evolution of life history traits in Rhinocerotoidea has been relatively conservative since the Oligocene and does not consistently correlate with shifts in body size. This study establishes a foundation for understanding the evolution of life histories in this group.

**Supplementary Information:**

The online version contains supplementary material available at 10.1038/s41598-025-13365-w.

## Introduction

Life history is a significant aspect of mammalian biology, offering valuable insights into their evolution^1–3^. For fossil species, teeth are among the most commonly used materials for inferring life history traits^4–10^. Kirillova and coauthors^11,12^ examined the age of death in two fossil rhinocerotids *Coelodonta* and *Stephanorhinus* from the Pleistocene, suggesting that their lifespans were comparable to those of extant African rhinocerotids. Recently, Lu et al.^13^ estimated the reproduction traits of the fossil rhinoceros *Plesiaceratherium* from the Early Miocene, noting that its age of body maturity was less than 10 years, earlier than that of modern African rhinocerotids. This raises the question: did earlier fossil rhinocerotoids, such as those from the Paleogene, exhibit distinct life history patterns?

The family Paraceratheriidae is renowned for its immense body sizes and specialized rostral region^14–17^. We present here a new species of *Turpanotherium* based on a new mandible from the Early Oligocene of Qingshuiying locality, Northwest China—a site renowned for its significant mammalian fossil finds, particularly rhinocerotoids^18–20^. This study will describe the morphological features of the newly discovered mandible, contributing valuable evidence for understanding tooth specialization within Rhinocerotoidea. Furthermore, we investigate whether the life history traits of this medium-sized Paleogene giant rhino, *Turpanotherium*, resemble those of Neogene or Quaternary Rhinocerotidae. This comparative analysis will unlock unique insights into the evolution of life history in Rhinocerotoidea.

**Fig. 1:**
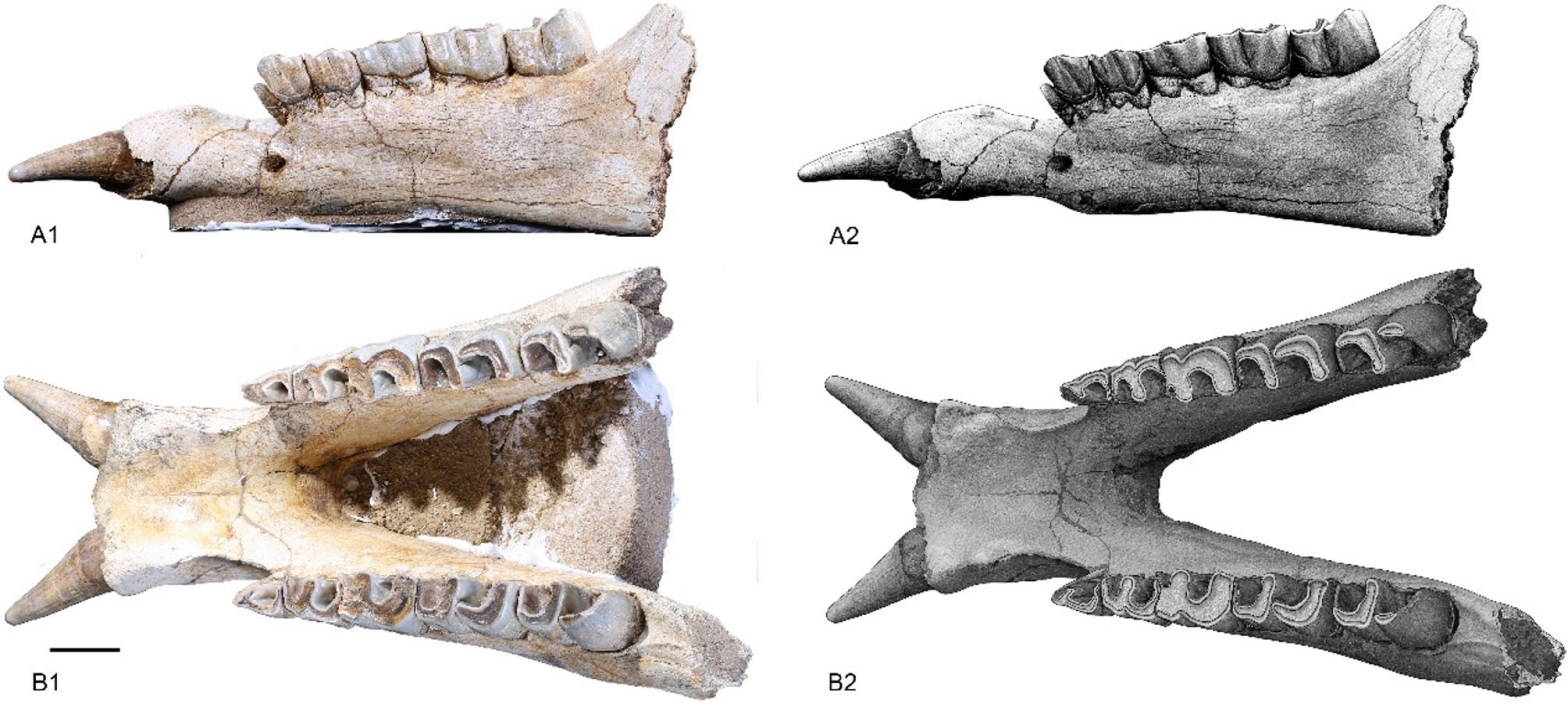
New mandible (IVPP V 33890) of *Turpanotherium qiui* sp. nov. from the Early Oligocene Qingshuiying Formation, Ningdong locality, Northwest China. A, right view; B, occlusal view. Scale bar = 50 mm.

## Result

### Systematic paleontology

Perissodactyla Owen, 1848.

Rhinocerotoidea Gray, 1825.

Paraceratheriidae Osborn, 1932.

*Turpanotherium* Qiu et Wang, 2007.

Type species: *Turpanotherium elegans* Qiu et Wang, 2007.

Include species: *Turpanotherium*? *yagouense* (Qiu et al., 2004); *Turpanotherium qiui* sp. nov.

Horizontal and locality: So far known from Turpan Basin of Xinjiang, Lanzhou and Linxia Basins of Gansu, Lingwu Basin of Ningxia, China. From the Early Oligocene to the Late Oligocene.

Diagnosis (revised from Qiu and Wang^15^): Size slightly smaller than *Aralotherium prohorovi*. Symphysis and i1 extend anteriorly, with their lower borders forming an almost straight line. A diastema is present between left and right i1s. Cheek teeth rather high-crowned. Both p1 and p2 gradually lost, and crowns of unworn p3 and p4 higher than long. Lunar with anterior “hindering facet”, two distal facets are unequally developed, with distal angle shifted strongly medially. Angles of PhIII’s are strongly reduced.

*Turpanotherium qiui* sp. nov.

(Fig. 1 and S1-S7, table S1)

Holotype: IVPP V 33890, a mandible with all lower teeth, but missing the ramus part.

Horizontal and locality: The fossil was directly exposed on the ground surface, and the surrounding rock is the low cemented yellow sandstone, with occasional small gravels. The Qingshuiying Formation, the Early Oligocene, Ningdong locality of Lingwu County, Ningxia, China.

Diagnosis: The symphysis posteriorly extends to level of p4. The ventral edge of the mandible is straight until the symphysis, which is slightly upturning. The p2 is present and columnar, with a single root. The trigonid of p3 is reduced in length and much shorter than that of its talonid. Lingual cingulua are strong in p3-p4, but gradually reduced from m1 to m3. This species is slightly smaller than *T. elegans* in size of cheek teeth.

**Fig. 2:**
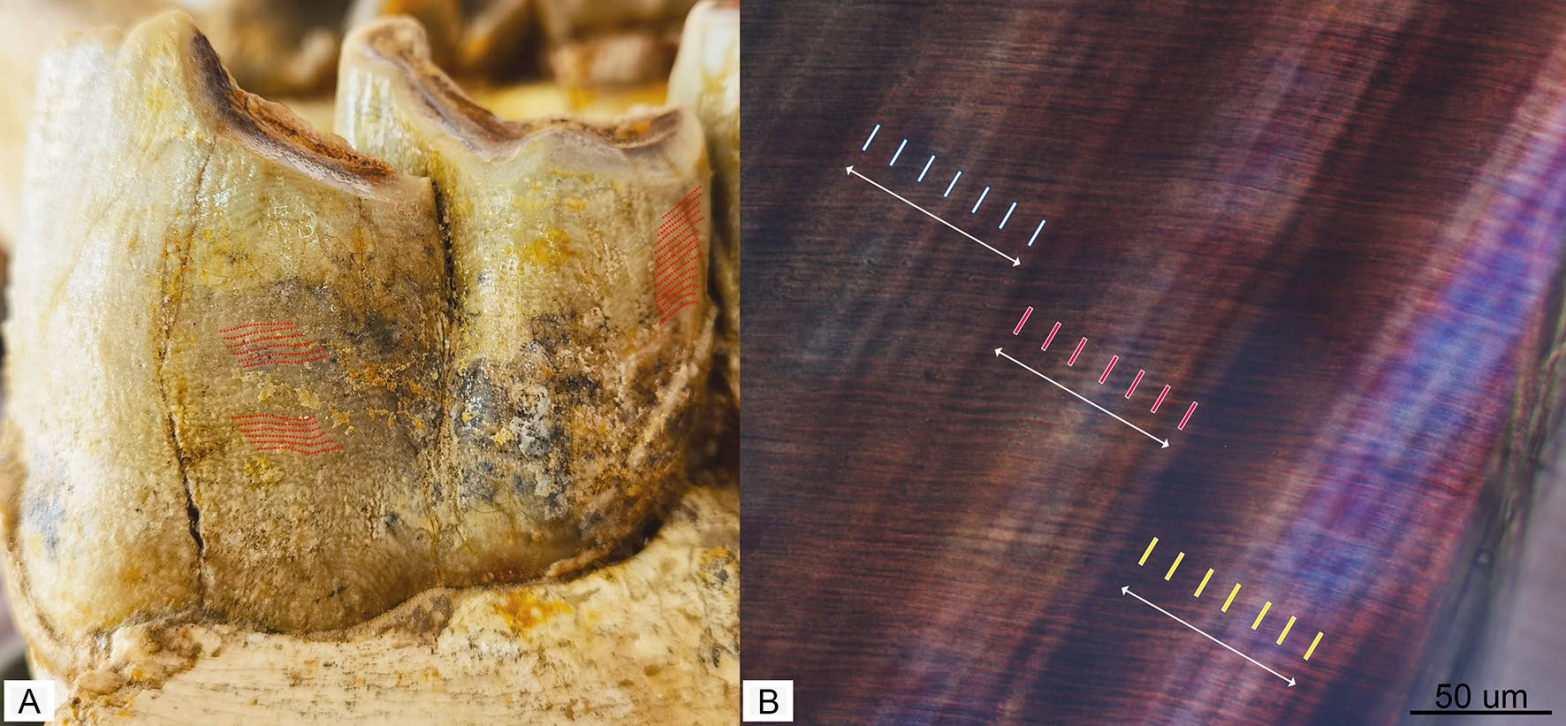
Longitudinal section of m1 of *Turpanotherium qiui* sp. nov. from the Early Oligocene Qingshuiying locality, Northwest China. A, labial view of m3, red dot-lines show the perikymata. B, three Retzius lines in enamel under polarizing microscope are marked, and enamel prisms are tightly stacked together. Each perikymata is corresponding to a Retzius line. The number of perikymata in m3 is 91, indicating a crown formation time 637 days at least, because of the highest cusp has been worn. Given all cheek teeth p3-m3 share the crown height, we suggested they may need the same time to form the crown enamel.

### Morphology description

The mandible is well-preserved but missing the ramus part. The ventral edge of mandible is nearly horizontal. The mental foramen is adjacent to the level of p2. The symphysis is not upturning at anterior part. In dorsal view, the posterior end of symphysis is at level of posterior part of p3. Anterior to p3, the symphysis shows a tendency of widening, and a pair of incisors follow this tendency and divergent anteriorly. The diastema between the first incisors i1 and the second lower teeth p2 is long. The canines and other incisors are completely reduced. The outline of i1 is cylindrical and tapering anteriorly, and it has a round and blunt tip. There has no cingulid surrounding the base of crown of i1.

In this specimen, dp1 (or p1) is reduced, the first cheek teeth present in the mandible are p2, which has a rounded outline in both crown and root. Cingulum surrounding the crown base of the left p2 is strong and form a pillar at the posterior-lingual corner. The paralophid is absent and make p3 an elongated triangular outline. The paraconid is also weak and lower than the protoconid, which is the strongest cusp of p3. The ectoflexid is narrow and extends downward to the labial cingulum. Anterior to the ectoflexid is another narrow groove extending through the crown tip to the labial cingulum. Both the labial and lingual cingula are continuous and strong. Both the paralophid and hypolophid extends straightly forward, and their labial walls are all slightly convex and rounded. The entolophid is much narrower than other lophids, and the entoconid is also weak. The entolophid extending transversely. The occlusal outline of the posterior valley is U-shaped. In p4, the special feature lies in the paralophid, which is strong and forming a transformed U-shaped anterior valley. The lower molars m1 and m2 and close to p4 in many aspects, but its labial cingulum is much weak. The crown of m3 is also similar to m1 and m2 but its entolophid show a tendency of inclining forward more than that of the metalophid, forming a shallow crescent outline with the hypolophid. The crown of m3 is at slightly wear stage, and the crown height measures 63.4 mm, approaching the occlusal length (67.6 mm), nearly a hypsodont.

**Fig. 3:**
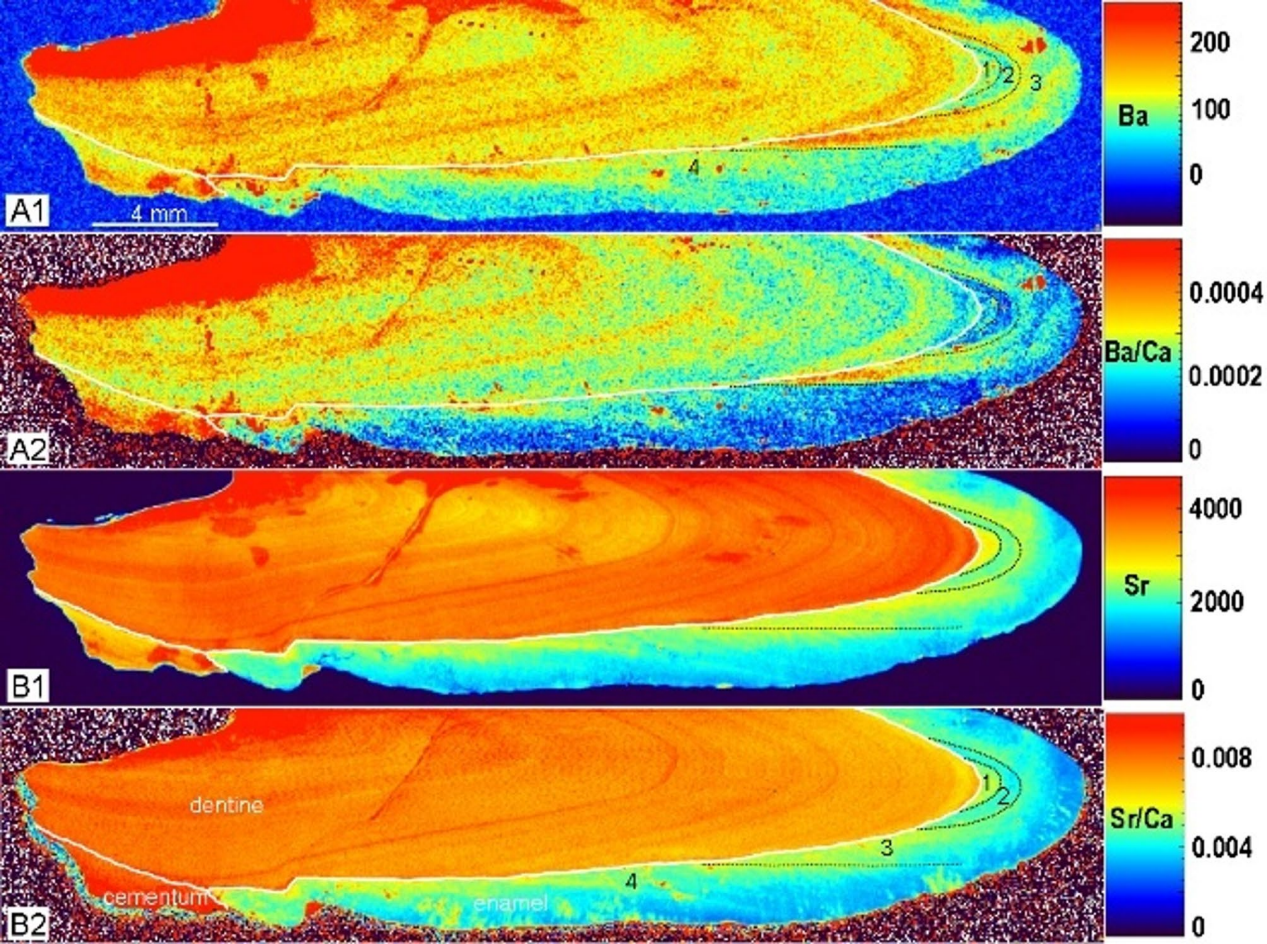
Elements concentration in enamel of p2 of *Turpanotherium qiui* sp. nov. from the Early Oligocene Qingshuiying Formation, Ningdong locality, Northwest China. A1, Ba; A2, Ba/Ca; B1, Sr; B2, Sr/Ca. Boundaries between enamel, cementum, and dentine are marked by white lines. Each element mapping is rendered at different concentration scales (not shown). With colors turning from warm to cool, element concentrations are decreasing. In this section, the concentration signal becomes weak under the enamel surface due to alteration during the fossilization process. Three distinct bands are recognized and marked by three black dot-lines, and each representing a continuous period of growth and characterized by varying elemental concentrations: 1, prenatal phase, Ba and Sr have higher concentrations; 2, neonatal phase, depositions of Ba and Sr become lower, indicating an intense stress response to both female and its calf due to birth; 3, breastfeeding period, namely weaning transition period, because vegetable food generally contains lower level of Sr and Ba than those of milk, so there is a fluctuating level of concentration. Duration of Ba concentration in the phase 3 is shorter than that of Sr, reflecting their different condition of concentration.

**Fig. 4:**
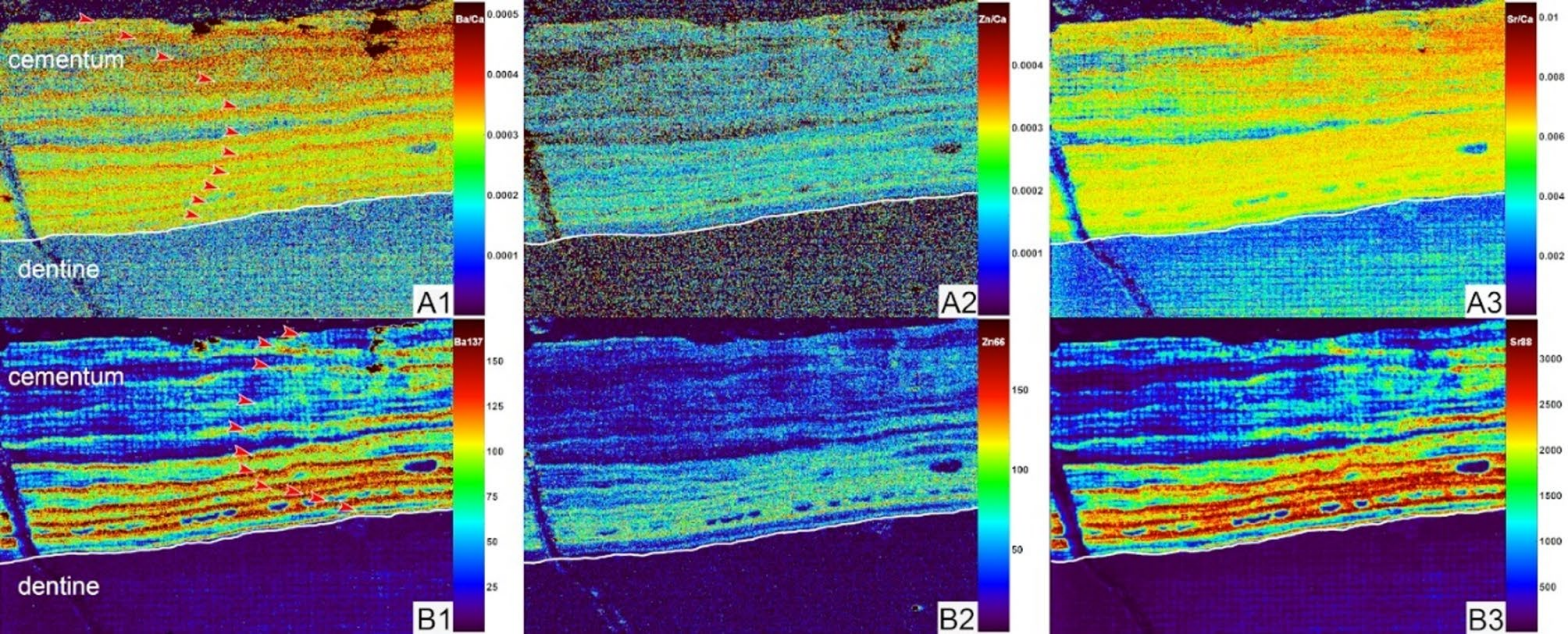
Elements concentration in root cementum of p2 of *Turpanotherium qiui* sp. nov. from the Early Oligocene Qingshuiying Formation, Ningdong locality, Northwest China. A1, Ba; A2, Zn; A3, Sr; B1, Ba/Ca; B2, Zn/Ca; B3, Sr/Ca. Boundaries between cementum and dentine are marked by white lines. Each element map is rendered at different concentration scales (not shown). With colors turning from warm to cool, element concentrations are decreasing. Totally eleven annual incremental lines are recognized, and marked by red arrowheads. Details of cementum with these lines under the polarizing microscope are available in Supplementary Figure S3.

### Biological traits

Two sections of p2 were prepared: the crown section, which was nearly sagittally separated from the rest of the tooth during excavation, and the section encompassing both the crown and root. The m1 sections were obtained from the crown-root junction area between the trigonid and talonid, where the cementum layer is thickest. Longitudinal sections of p2 and m1 revealed well-exposed enamel and dentine within the crown. Retzius lines were clearly visible, with seven daily cross-striations per line (Fig. 2). In the enamel section of p2, an area without visible or countable incremental lines was observed, limiting the accurate estimation of its crown formation time. Despite this limitation, the p2 section clearly displayed 61 Retzius lines throughout the enamel, indicating a minimum crown formation time of 427 days for p2 (Figure S6). Although the highest point of the m3 crown is slightly worn, 91 perikymata were identifiable at the hypoconid position. Considering that the protoconid is generally the highest point of lower cheek teeth in rhinocerotoids, the minimum crown formation time of m3 is estimated to be 637 days (Fig. 2).

The enamel section of p2 revealed four prominent bands in the barium and strontium element mapping, with slight variations in the third and fourth bands (Fig. 3). At the crown tip’s innermost region, a crescent-shaped area of prenatal enamel was evident, characterized by higher levels of strontium and barium. This was followed by a second crescent-shaped area, the neonatal region, marked by relatively lower strontium and barium concentrations. This short period suggests a rapid transition from prenatal to postnatal life. The third area is the breastfeeding period, with strontium and barium levels similar to that of the first. However, the barium concentration showed a relatively narrower third band compared to the strontium band, consistent with previous studies suggesting that strontium displays variable concentration patterns during the weaning period (e.g., increase, decrease, or delayed changes)^21,22^.

We also considered the potential impact of seasonal fluctuations in element concentrations on the enamel record, which may overlap with dietary shifts. Two distinct lines of elevated strontium and barium concentrations are visible in the enamel (Figure S4): one at the boundary between the third and fourth bands, and the another within the fourth band. Given the relatively short enamel formation time between these two lines, it is unlikely that both are solely attributable to seasonal variation. A more plausible interpretation is that the first line marks the weaning event, while the second remains uncertain, likely reflecting a seasonal, or disease, or other abnormal influence characterized by elevated barium and strontium concentrations.

Austin et al.^23^ observed concentration differences in human teeth between periods of exclusive breastfeeding and weaning transition. Similarly, in our study, barium concentrations continued to fluctuate even beyond the third band period. Considering the approximately 1-3-year breastfeeding period observed in extant rhinoceroses, we propose that the third band represents a period of exclusive breastfeeding, while the fourth band marks a transition period. Based on this, we estimate a minimum suckling duration of 427 days for this giant rhino.

**Fig. 5:**
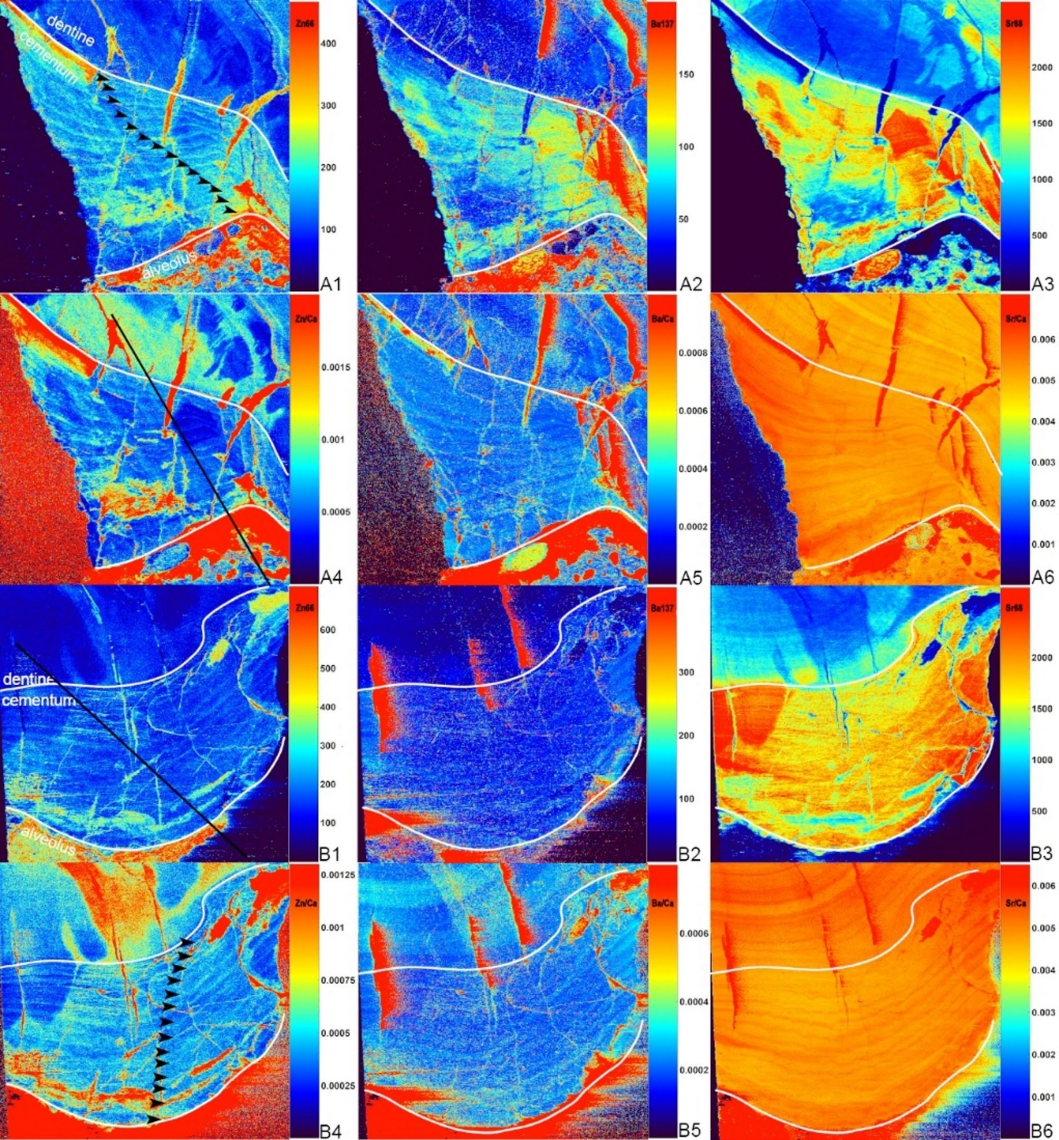
Elements concentration in root cementum of m1 of *Turpanotherium qiui* sp. nov. from the Early Oligocene Qingshuiying Formation, Ningdong locality, Northwest China, showing the annual incremental lines. A, lingual part; B, labial part. 1, Zn; 2, Ba; 3, Sr; 4, Zn/Ca; 5, Ba/Ca; 6, Sr/Ca. Each elemental mapping is rendered at different concentration scales (not shown). With colors turning from warm to cool, element concentrations are decreasing. Cementum, dentine, alveolus are bounded by white lines. Ratio mappings have clearly bounded incremental lines than each singe elemental map. Totally fourteen lines are counted in cementum of both sides.

Both barium and zinc exhibit distinct annual increments in the acellular cementum surrounding the roots of p2 and m1 (Figs. 4 and 5). Strontium concentration is significantly higher than those of barium and zinc. The total number of incremental lines were counted 14 in m1 and 10–11 in p2. Given that the crown formation time of m1 is around 2 years, the estimated body maturity age of this individual is at least 17 years old.

There is one point have to be mentioned that the zinc concentration has been used to indicate the neonatal line in section of teeth. In human, the birth line of M1 enamel shows a lower zinc concentration relative to prenatal and postnatal periods^21^. By contrast, in the Mesozoic eutherians, the birth line has a higher zinc concentration than the prenatal and postnatal periods^7^. In this study, in section of p2 the element zinc does not show the fluctuation with birth and weaning whatever using methods of LA-ICP-MS or LIBS (Figure S7). Based on this weak information, calculation of the chronological time is very difficult and not reliable relative to information of barium and strontium. Nevertheless, above interpretations about birth and weaning time of new material are beyond question, because the evidences of barium and strontium are strong.

### Taxonomic identity

The newly discovered mandible exhibits several diagnostic features: a pair of large, horizontally implanted first lower incisors (i1) with all other incisors and the canines completely reduced; a single-rooted p2 with a cylindrical rather than triangular outline; and both the protolophid and hypolophid of the lower cheek teeth extending nearly sagittally. These characteristics differentiate this specimen from most Paleogene rhinocerotoids. For instance, Hyracodontidae retain all incisors and canines, with significantly smaller overall body sizes; most members of Rhinocerotidae possess a pair of second lower incisors (i2) but lack other incisors and the canine; and Amynodontidae exhibit unspecialized incisors along with a pair of enlarged canines^14^.

The only rhinocerotoid resembling this mandible in its diagnostic features is the medium-sized giant rhino *Turpanotherium*, within the family Paraceratheriidae. The new mandible shares similarities with the type species *Turpanotherium elegans* in several aspects, such as the enlarged second lower incisors i2; the wide distance between i2 on both sides; the longer diastema between the cheek teeth row and the root of i2. This new mandible differs from *T. elegans* by the presence of p2, a tooth typically lost in *Turpanotherium*. Previously, genera such as from the Eocene retained both the first and second lower premolars within Paraceratheriidae. In *Paraceratherium*, the crown of p2 resembles *Juxia* and *Urtinotherium* morphologically but exhibits a single root^15,17^. *Turpanotherium* lost its second premolar in the type species *T. elegans*^15^. The newly discovered mandible of *T. qiui* reveals a single-rooted p2 characterized by a cylindrical contour extending from the root to the crown, with a complete absence of typical cheek tooth morphology^15^. The mandibular symphysis of the new specimen exhibits a slightly concave ventral border and convex dorsal border, contrasting with the nearly straight ventral and dorsal borders observed in *T. elegans*. The interincisal angle measures 83° in the new mandible, compared to 92° in *T. elegans*. Regarding dental wear patterns, the new specimen shows a p3 crown height exceeding that of p4 at its current wear stage, whereas this relationship is reversed in *T. elegans* (p4 > p3 in crown height).

The exceptional length of the incisor in the new mandible (88 mm crown axis length) warrants further attention, as it significantly exceeds that of *T. elegans* (48 mm). The enlargement of lower incisors within Paraceratheriidae began during the Eocene with *Urtinotherium* and culminated in both *Turpanotherium* and *Aralotherium*. The longer incisor may be considered a more advanced feature. However, further analysis reveals that *T. elegans* possesses a longer and more retracted symphysis compared to the new mandible. Specifically, the posterior end of the symphysis in *T. elegans* extends to the middle of p4, whereas in the new mandible, it reaches only the level of p3. Additionally, the diastema distance between p3 and the i1 root in *T. elegans* (40 mm) is similar to the distance between p2 and the i1 root in the new mandible (40 mm). Sexual dimorphism in specialized teeth and skull horns is well documented within various rhinocerotoid families, such as the canines in Amynodontidae and the i2 incisors in Rhinocerotidae^14,24–26^. For instance, in *Aralotherium prohorovi*, the crown size of i1 ranges from 37 mm to 55 mm^27^. Therefore, we suggest that the shorter i1 in the holotype mandible of *T. elegans* probably represents a female individual or a lower value of length range. The male counterpart, with a larger i1, would likely exhibit a corresponding longer symphysis to accommodate the larger tooth.

Based on these observations, we confidently designate the new mandible as a distinct form of *Turpanotherium*, designated as *Turpanotherium qiui* sp. nov. in honor of Dr. Qiu Zhan-Xiang, whose significant contributions to Cenozoic mammalian evolution and stratigraphy have greatly enriched our understanding. Given the presence of p2, *T. qiui* sp. nov. represents a more primitive morphology than *T. elegans*.

## Discussion

### Dentition development

Dentition development is closely correlated with life history pace^4–8^. The eruption sequence of teeth in rhinocerotoids has been remarkably conserved throughout their ~ 60 million years of evolution, and the newly discovered mandible conforms to this pattern, exhibiting the sequence m1-m2-p2/p3-p4-m3 ^13^. Based on the lamination lines of enamel, namely the perikymata and Retzius lines, the crown formation time of m3 of *Gaindatherium* (Rhinocerotidae) from the Late Miocene Siwalik, Pakistan, is 651–721 days; and, the time of M1 of the extant rhinoceros *Rhinoceros unicornis* is 574–714 days^28^. In this study, the crown formation time of m3 of *T. qiui* sp. nov. is at least 637 days. On the other hand, the crown height of lower molars of *Turpanotherium* is up to 60 mm, similar to those of most latterly appeared rhinocerotoids from the Neogene and Quaternary^15,29^. Furthermore, in the new mandible, the crown height of m3 measures 63.4 mm, approaching the occlusal length (67.6 mm). This tooth m3 is slightly worn, meaning its current height does not represent the maximum original crown height. Therefore, the crown height-to-length ratio was likely ≥ 1 when unworn. Based on established hypsodonty indices^30^, we suggest this specimen likely possessed hypsodont dentition.

The number of incremental lines in the m1 cementum of the new mandible, where the third molar (m3) is at slightly wear stage, falls within the range observed for age class XIII (11–15 lines, 14–18 years) in *D. bicornis* and age class XII (14 ± 2.6 lines, 14–20 years) in *C. simum*^31,32^. In extant black rhinoceros *Diceros bicornis* and white rhinoceros *Ceratotherium simum*, the first molar (m1) erupts at approximately three years of age. When applying this observation to the new mandible, with its ten and fourteen incremental lines in the p2 and m1 cementum, respectively, we can estimate its age to be 17 years old, which aligns closely with the range observed in extant African rhinoceroses. Additionally, an individual of *Coelodonta* from a thermokarst lake in the Bol’shaya Chukoch’ya River basin in northeastern Republic of Sakha (Yakutia), Russia, exhibits a deeply worn M1 and is estimated to be 31–32 years old^11^. Similarly, a *Stephanorhinus* discovered in the Chondon River valley, Arctic Yakutia, Russia, with a slightly worn crown of M3, is estimated to be 20 years old^12^.

### Lifespan

Lifespan is closely correlated to body size and many studies have established equations to model this relationship^33,34^. The reconstructed mandible of *T. qiui* measures 665 mm in length, with an estimated skull length 800 mm, slightly larger than those of *Coelodonta*, *Stephanorhinus* and the extant African rhinoceros *Ceratotherium simum*^35^ (Guérin, 1980). Based on incremental line analysis of wild specimens, the oldest recorded ages at death for extant African rhinoceroses *Diceros bicornis* and *C. simum* are 37 years and 40 years, respectively. In captivity, *Dicerorhinus sumatrensis* has been documented to live up to 32 years at the Cincinnati Zoo in Ohio, USA. Similarly, *Rhinoceros unicornis* can live up to 40 years, while *D. bicornis* and *C. simum* can exceed 50 year^36^. Fossil evidence suggests that *Coelodonta* could live up to at least 31 years^11^. Given the similarities in dental eruption schedule and body size, it is reasonable to postulate that *Turpanotherium* could also live up to 40 years or even longer. These age records are roughly in concordance with the equation computing based on body size^37^.

*Plesiaceratherium* from the Early Miocene presents an intriguing case study of the relationship between body size and life history. Over evolutionary time, the body size of perissodactyls has increased significantly^38^. Although *Plesiaceratherium* shares similarities in crown height, body size, and weaning and sexual maturity ages with the aforementioned Quaternary groups, it exhibits a notably earlier age of body maturity, reaching maturity before 10 years of age^13^and probably had a shorter lifespan. This suggests that, unlike these genera, the lifespan of *Plesiaceratherium* did not proportionally elongate with its increasing body size. In other words, for *Plesiaceratherium*, the evolution of larger body size preceded the evolution of elongated life history traits. Another example is *Elasmotherium*, which shares a similar body size with *Turpanotherium* but possesses much higher crown height in its cheek teeth, namely the evolution of crown height of teeth preceded the evolution of the body size. These cases underscore the complexity of life history evolution, which does not always correspond directly with changes in body size.

## Conclusion

The newly discovered mandible from the Early Oligocene Qingshuiying Formation, Ningdong locality in Northwest China is designated as *Turpanotherium qiui* sp. nov. This species is more primitive than the type species *T. elegans* due to the presence of the second lower premolar (p2). Based on the elongated lower incisor, we postulate that this mandible represents a male individual. Reductions in incisors and premolars within Paraceratheriidae are accompanied by high-crowned teeth and large body size, with *Turpanotherium* representing the most advanced stage of tooth specialization within Rhinocerotoidea during the Oligocene.

The analysis of p2 and m1 sections provides valuable insights into age estimation. Based on the estimated crown formation time of m3 at 637 days, other cheek teeth with comparable crown heights are likely to follow a similar developmental timeframe. Incremental lines in the enamel and cementum chronicle key life history events, revealing a lactation period of at least 427 days, an age of body maturity around 17 years, and a potential longevity of up to 40 years. This life history pace is comparable to that of Quaternary Rhinocerotidae. These findings suggest that the evolution of life history within Rhinocerotoidea has been relatively conservative, with its maximum potential values established during the Oligocene.

Comparisons with known records of Rhinocerotidae indicate that while body size is closely correlated with life history, the two are not always congruent. The complexity of life history evolution suggests that, beyond body size, additional factors must be considered when reconstructing the life histories of fossil species in future research.

## Method and materials

### Morphological description

This study describes a new mandible from the Early Oligocene of the Ningdong locality, Ningxia, China. The specimen preserves the horizontal portion of the mandible but lacks the ramus. Notably, it retains a pair of incisors and two complete cheek tooth rows (p2-m3) in excellent condition. A detailed description, measurement, and comparison of this mandible with other rhinocerotoid specimens were conducted. The terminology for the mandible and teeth used in this study follows the framework established by previous work^15^and dental measurements were performed according to the protocols outlined by Guérin^35^.

### Preparation of section

In this study, the second lower premolar (p2) and the first molar (m1) of the new mandible (IVPP V 330089) were selected for sectioning. At their developmental stage, these teeth offered the greatest potential for age estimation due to their early eruption and the maximum number of recorded incremental lines. Sectioning was conducted following established protocols in the laboratory of the Institute of Vertebrate Paleontology and Paleoanthropology (IVPP), Chinese Academy of Sciences (CAS)^13^. Two sections of p2 were prepared: the crown section of the labial part, which was nearly sagittally separated from the rest of the tooth during excavation, and the section of the remaining lingual portion encompassing both the crown and root. The m1 sections were obtained from the crown-root junction area between the trigonid and talonid, where the cementum layer is thickest. Detailed section positions are provided in the Supplementary Figures S1 and S2. Initial examinations of these sections were performed using a ZEISS optical microscope equipped with polarized light.

### Incremental lines

Tooth crown development begins at the tip of the dentine crown, involving regular enamel secretion and mineralization. The incremental features of the tooth crown are recorded in the enamel as short-term daily laminations and long-term periodic Retzius lines, both of which are observable in sections under a polarizing microscope^8^. Perikymata, a type of regularly occurring imbricational striae surrounding the enamel surface of the crown, corresponds rhythmically to the Retzius lines.

Once the interval of Retzius lines is determined using a polarizing microscope, crown formation time can be calculated by counting either the perikymata or the Retzius lines in the enamel. Due to wear, not all lines or perikymata are well-preserved or fully exposed. From the crown tip to the base, the widths of individual Retzius lines and perikymata varies slightly. Based on these features, the crown formation time of the m3 in *Gaindatherium* (Rhinocerotidae) is estimated to be 651–721 days, while the formation time of the M1 in *Rhinoceros unicornis* ranges from 574 to 714 days^28^.

Cementum is a mineralized connective tissue deposited around the crown or root surfaces of mammalian teeth. As part of the tooth anchorage system, it serves to attach the tooth to the jawbone by connecting the periodontal ligament to the dentine surface^39^. Incremental lines in the cementum surrounding the dental root are widely recognized as a reliable index for age determination in several living mammals^40–42^. Furthermore, this index has been shown to align closely with dental development and attrition throughout the life history of African rhinoceroses^31,32^. Using cementum from cheek teeth, the ages at death of Quaternary fossil rhinocerotids *Coelodonta* and *Stephanorhinus* have also been successfully estimate^11,12^.

### Analysis of elements concentrations

Element concentrations within the sections were analyzed using both laser-induced breakdown spectroscopy (LIBS) and laser ablation–inductively coupled plasma–mass spectrometry (LA-ICP-MS), following established protocols^21,23,41,43–46^. Parameters of both methods LIBS and LA-ICP-MS are provided in the Supplementary Tables S2-S4). The enamel image of the longitudinal section of p2 was obtained using LIBS. The concentration distribution of zinc in the thin section of p2 shows no seasonal variation and is provided in the Supplementary Figure S7. The cementum of both p2 and m1 was analyzed using LA-ICP-MS. Additional images of the section positions for elemental analysis are available in the Supplementary Figures S3, S4 and S5.

### Preparation of section

In this study, the second lower premolar (p2) and the first molar (m1) of the mandible were selected for sectioning. At their developmental stage, these teeth offered the greatest potential for age estimation due to their early eruption and the highest number of recorded incremental lines. Sectioning was performed following established protocols in the laboratory of the Institute of Vertebrate Paleontology and Paleoanthropology (IVPP)^13^. The p2 sections were divided into two parts: the labial crown, which became separated from the rest of the tooth during excavation, and the remaining lingual portion, encompassing both the crown and the root. The m1 sections were obtained from the crown-root junction area between the trigonid and talonid, where the cementum layer is thickest. Further details on sectioning positions are provided in the supplementary materials. These sections were initially examined using a Zeiss optical microscope equipped with polarized light.

## Supplementary Information

Below is the link to the electronic supplementary material.

Supplementary Material 1

## Data Availability

The datasets generated and/or analysed during the current study are available as “Supplementary materials”.
