# Supplementary material for: Life history of a new Paraceratheriid from the Early Oligocene of Northwest China
Source: Sci Rep. 2025 Aug 6;15:28740. doi: 10.1038/s41598-025-13365-w (PMC12328695; doi:10.1038/s41598-025-13365-w)
Supplement: Supplementary file 1 — Supplementary Material 1 [file 41598_2025_13365_MOESM1_ESM.docx]

Supplementary information

1. Supplementary tables

Table S1. Measures of teeth of *Turpanotherium lingwuensis* sp. nov. and other rhinoceroses (length/width/height) (mm).

|  | *T. lingwuensis* | *T. elegans* | *J. sha* | *Urt. inter* | *A. prohorovi* | *A. sui* | *P. linxiaense* | *P. grangeri* |
| --- | --- | --- | --- | --- | --- | --- | --- | --- |
| p2 | 16.4/13.2/24.5 | - | 20.6/14/- | 32/24/- | 29.5-33/21.5-24.5/- | 39/31/- | -/-/- | 33/24/- |
| p3 | 37.1/23.9/46.6 | 38.3/25.5/- | 26.2/18.8/- | 43/35/- | 43-46/37-39/- | 57/48/- | 54/43/- | 51/37/- |
| p4 | 41.5/34.3/38.2 | 48.7/34.3/- | 24.6/20.5/- | 48/40/- | 55-63/44-50/- | 65/53/- | 66/50/- | 56/42/- |
| m1 | 54.9/38.1/35.4 | 56/47/- | 35/27.6/- | 60/45/- | 63-73.5/48-56/- | 81/55/- | 69/49/- | 77/55/- |
| m2 | 65.1/39.8/43.6 | 63.7/37.7/- | 35.5/28.5/- | 66/48/- | 71-83/50-60/- | 91.6/58 | 85/56/- | 92/60/- |
| m3 | 67.6/36.5/63.4 | 80.4/41.4/- | 36.8/26.8/- | 66/53/- | 72-96/50-60/- | 102/59/- | 91/55/- | 89/55/- |
| i1 | 88.4/45.8/38.9 | 48/35/- | 19.7/14.4/1.37 |  | 37-55/32-41/- | 73/55/- | 33/32/- | 45/39 |
| p2-p4 | 95 | - |  |  | - | - | 109 | 145 |
| m1-m3 | 194.9 | - |  |  | - | - | 249 | 254 |
| p2-m3 | 289 | - |  |  | - | - | 355 | 400 |
| width of symphysis | 140 | - |  | 100 | - | - | - | - |
| narrowest of symphysis | 121 | 123 |  | 90 | - | - | - | 95 |
| length of symphysis | 186.3 | - |  | 146 | - | - | - | 212 |
| distance i1-(p2) | 117 | - |  |  | - | - | - | 127 |
| distance (p2)-posterior  end of symphysis | 31 | - |  |  | - | - | - | - |

Data of *A. prohorovi* from Gromova, 1959; *A. sui* from Ye et al., 2003; *T. elegans* and *P. grangeri* from Qiu and Wang, 2007; *P. linxiaense* from Deng et al., 2021.

Table S2 Operating conditions for TOF-ICP-MS system

| TOF-ICP-MS |  |
| --- | --- |
| Nebulizer gas flow (L min^-1^) | 1.14 |
| Auxilary gas flow (L min^-1^) | 0.8 |
| Plasma gas flow (L min^-1^) | 16 |
| RF power (W) | 1400 |
| Detector | MCP |

Table S3 Operating conditions for LA-TOF-ICP-MS system (NWR 193ImageGEO)

| Laser ablation | |
| --- | --- |
| Wavelength (nm) | 193 |
| Pulsewidth (ns) | 5-10 |
| Ablation frequency (Hz) | 200 |
| Spot size (µm) | 60 |
| Laser energy (J/cm^2^) | 14.5 J/cm^2^ |
| Scan rate (µm s^-1^) | 12000 |
| Scan method | Image |

Table S4 Information of section and detecting method of incremental lines

|  | numbers | slice direction | detecting position | method | incremental lines |
| --- | --- | --- | --- | --- | --- |
| p2 | 1 | sagittal | crown enamel | polarizing-microscope | enamel Retzius’ line |
|  |  |  |  | libs | elements concentration |
|  | 2 | sagittal | crown | polarizing-microscope | enamel Retzius’ line |
|  |  | sagittal | root cementum | polarizing-microscope | cementum annual lines |
|  |  |  |  | top-icp-ms | elements concentration |
| m1 | 1 | coronal | root cementum (labial/lingual) | polarizing-microscope | cementum annual lines |
|  |  |  |  | top-icp-ms | elements concentration |

1. Supplementary figures

Following are figures and captions, which are cited in main text.


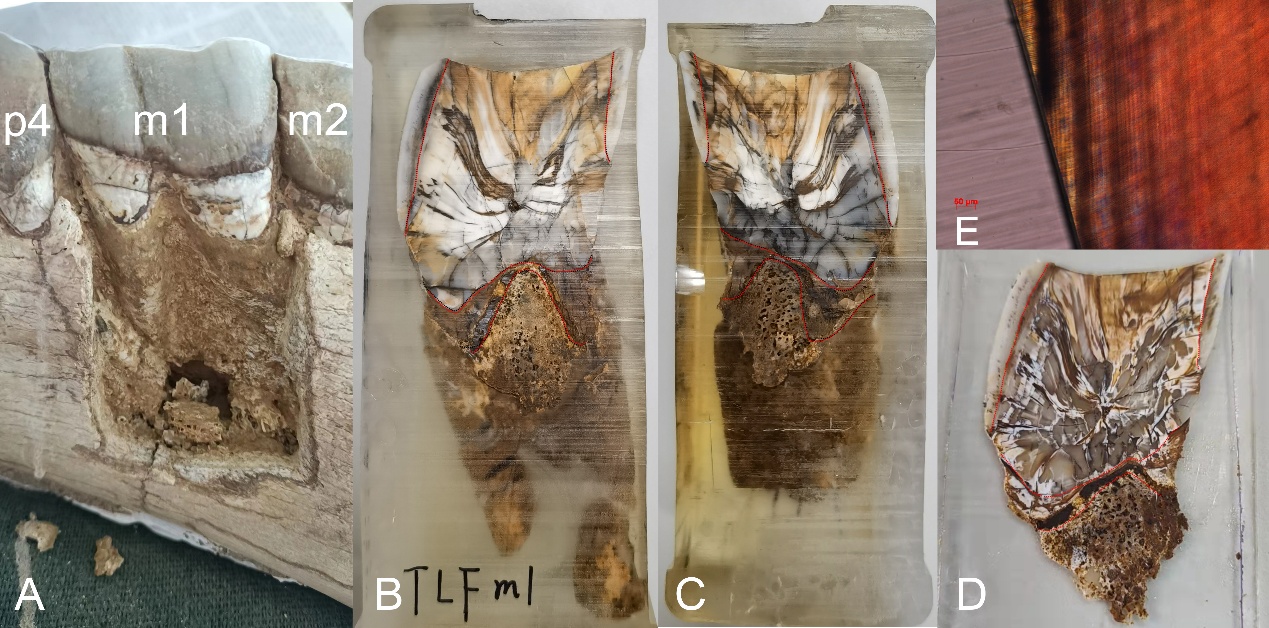


Figure S1. Preparing section of m1 of *Turpanotherium qiui* sp. nov. from the Qingshuiying locality of Northwest China. A, detaching m1 from the mandible; after imbedding in resin, m1 is dissected through joint region between trigonid root and talonid root, the red line indicates the coronal position of section; B is trigonid part, and C is talonid part; D, overview of finished section, which is detached from talonid part. E, the Retzius’ lines and cross striations of enamel in m1. Enamel, dentine, cementum, alveoli areas are delineated using dotted-lines with different colors.


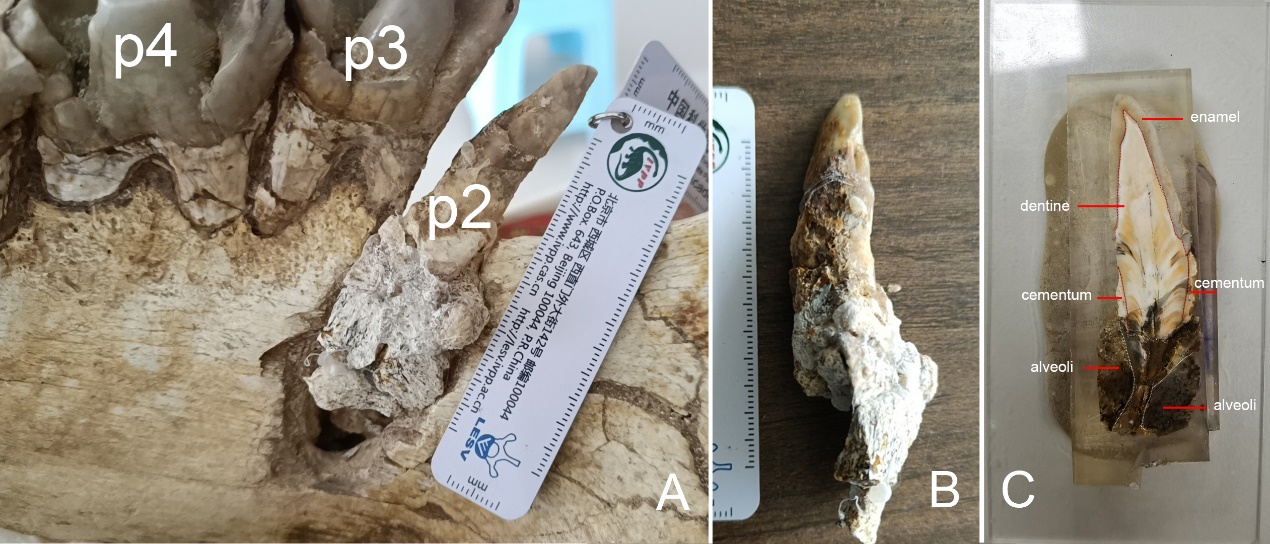


Figure S2. Preparing section of p2 of *Turpanotherium qiui* sp. nov. from the Qingshuiying locality of Northwest China. A and B, detaching p2 from the mandible; C, lingual part, exposed the tangent plane. Enamel, dentine, cementum, alveoli areas are delineated using dotted-lines with red color.


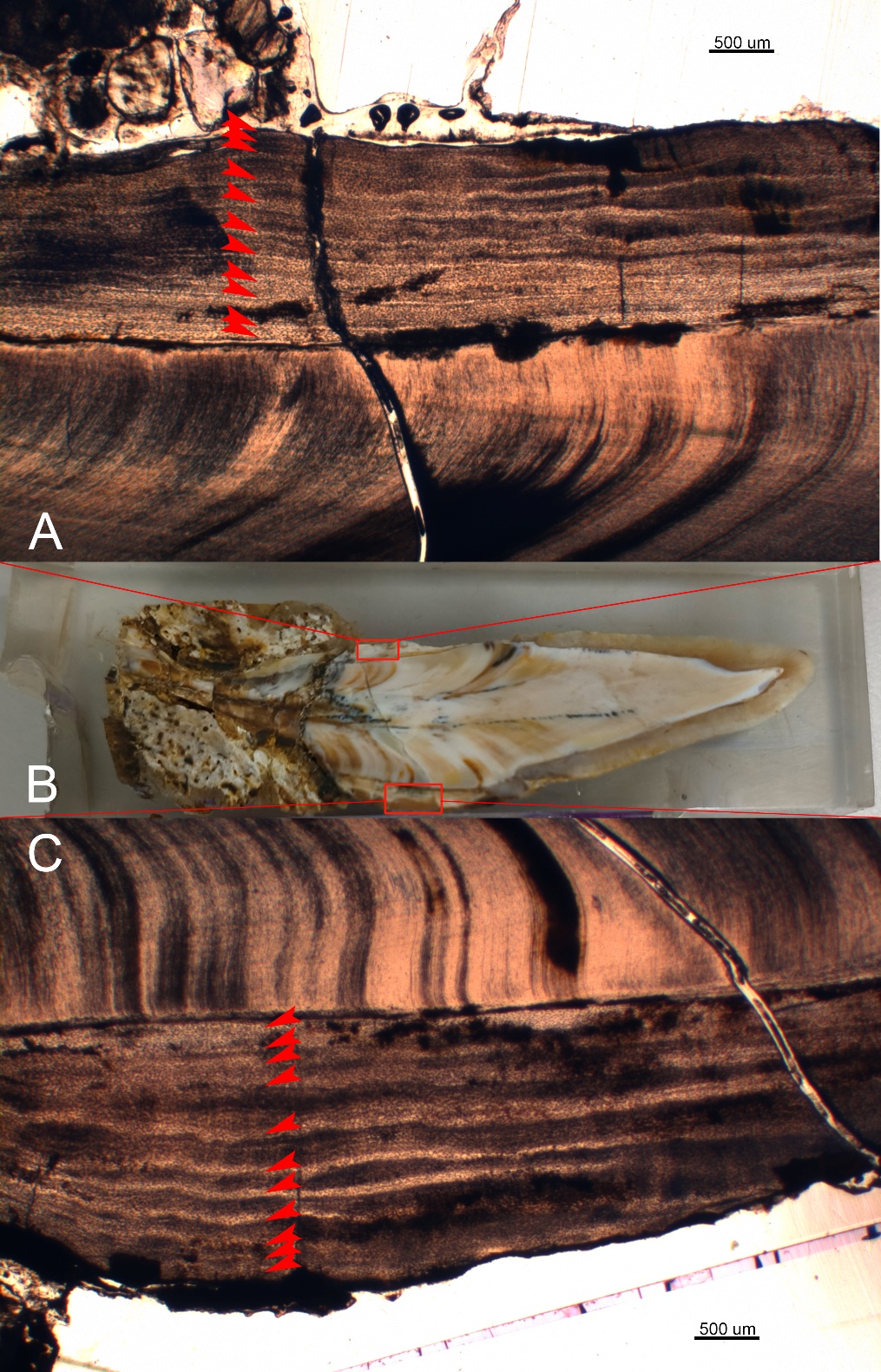


Figure S3. Section of p2 of *Turpanotherium qiui* sp. nov. from the Qingshuiying locality of Northwest China. B is overview of section of p2, and two red boxes indicate locations of cementum shown in A and C, where annual incremental lines are clear and marked by red arrowheads, which are counted eleven.


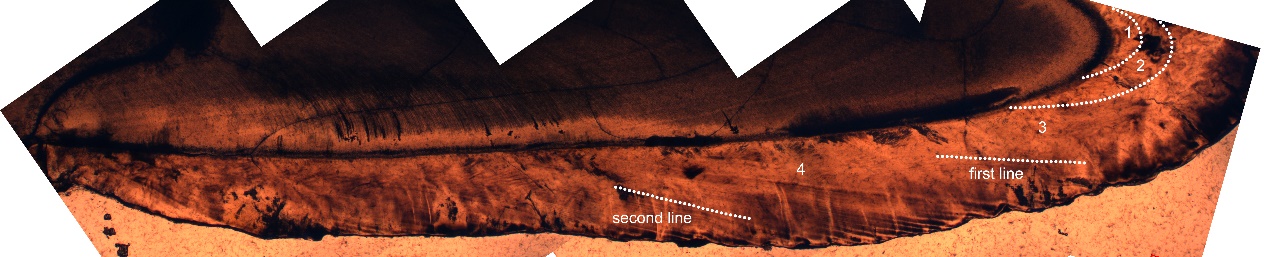


Figure S4. Combined overview of the first section of p2 of *Turpanotherium qiui* sp. nov. from the Qingshuiying locality of Northwest China. The birth line marked by the white dotted line. The second and third bands are not clearly bounded in this polarized light picture, their boundaries (white dotted line) are based on that of the element concentration. The first line and second line are marked with white dotted lines. And around the first line, the clear Retzius line begin to appear in the enamel.


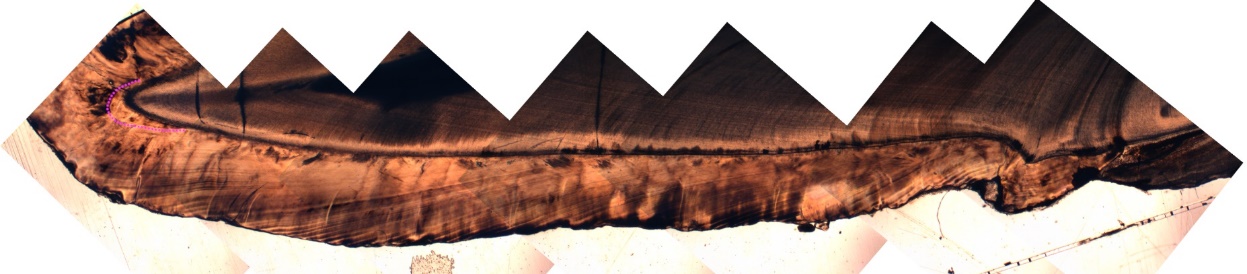


Figure S5. Combined overview of the second section of p2 of *Turpanotherium qiui* sp. nov. from the Qingshuiying locality of Northwest China. The birth line marked by the purple dotted line. Due to the sample position is different with the first section, the outline of birth line is slightly aslant.


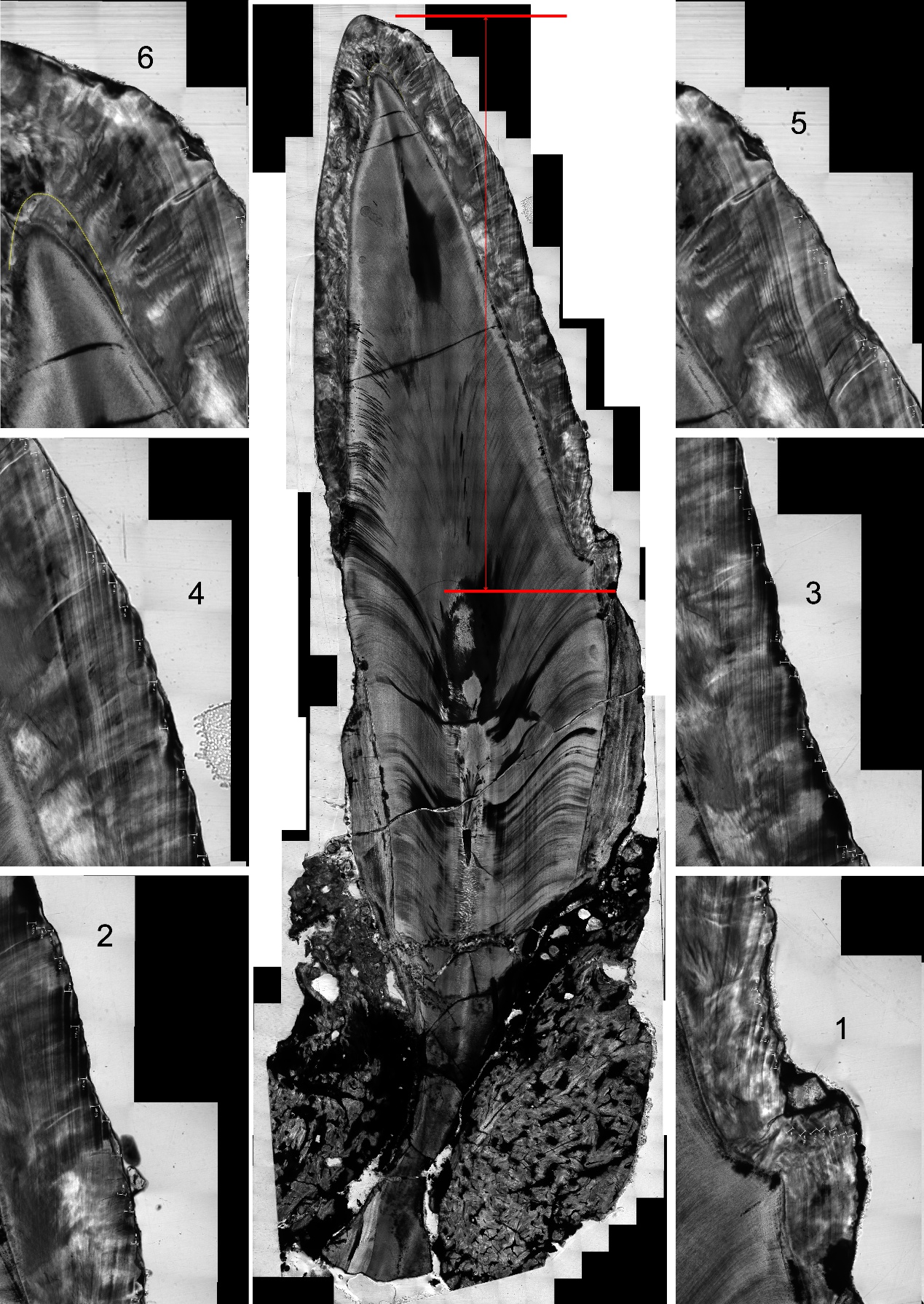


Figure S6. Overview of the second section of p2 of *Turpanotherium qiui* sp. nov. by scan microscope. Middle picture is overview, beside which are eight snapshots of counting the Retzius’ and lamination. The snapshots are captured using the LAS-AF-lite of LEICA company, and each counting unite is marked using white line with length signal. The six snapshots are numbered through crown basal to tip. The counting at basal part of crown, each counting unit (marked by white line) represent a Retzius’ line.


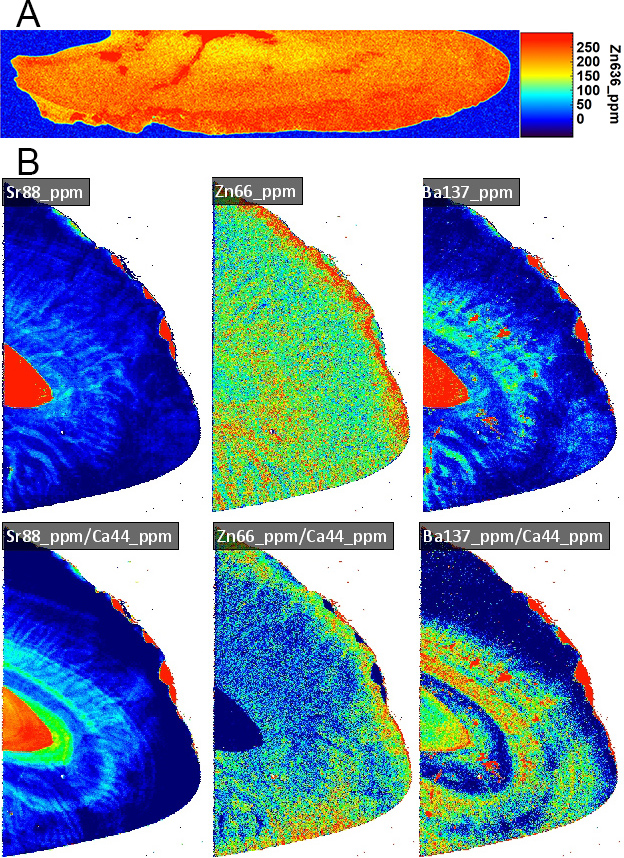


Figure S7. A, Zinc concentration of Figure 3, namely the crown section of p2, there has not clear variation through the section on either enamel of dentine. B, Zinc, Barium, and Strontium concentration at crown tip area of Figure S6, namely the second section of p2 with crown and root, using the method of LA-TOF-ICP-MS. At present, unlike Barium and Strontium, there has not notable regular zinc concentration corresponding to the birth and dietary change in the enamel.
